# Tumor burden score dictates prognosis of patients with combined hepatocellular cholangiocarcinoma undergoing hepatectomy

**DOI:** 10.3389/fonc.2022.977111

**Published:** 2023-01-23

**Authors:** Gang Deng, Jun-kai Ren, Hai-tao Wang, Liang Deng, Zu-bing Chen, You-wen Fan, Ya-jun Tang, Tong Zhang, Di Tang

**Affiliations:** ^1^ Department of General Surgery, the Seventh Affiliated Hospital of Sun Yat-sen University, Shenzhen, China; ^2^ Department of Hepatic Surgery and Liver Transplantation Center, the Third Affiliated Hospital of Sun Yat-sen University, Guangzhou, China

**Keywords:** combined hepatocellular-cholangiocarcinoma, tumor burden score, curative resection, overall survival, tumor relapse

## Abstract

**Background:**

The prognostic value of the tumor burden score (TBS) in patients with combined hepatocellular-cholangiocarcinoma (cHCC-CCA) remains unknown. This study aimed to investigate the impact of TBS on long-term outcomes after surgery.

**Methods:**

Patients who underwent radical-intent resection between June 2013 and December 2019 were retrospectively reviewed. Kaplan–Meier curves were used to analyze patient survival, and disease-free survival (DFS) and overall survival (OS) were examined in relation to TBS.

**Results:**

A total of 178 patients were included in this study, with 119 in the training cohort and 59 in the validation cohort. Kaplan–Meier curves showed that TBS was a strong prognostic indicator in patients with cHCC-CCA. Elevated TBS was associated with poorer DFS and OS (both P-value < 0.001) and was identified as an independent prognostic indicator. In addition, the prognostic value of TBS outperformed tumor size and number alone, microvascular invasion, and lymph node invasion. The prognostic significance of TBS was confirmed by the internal validation cohort.

**Conclusions:**

The present study suggested the significance of tumor morphology in assessing the prognosis of patients with cHCC-CCA who undergoing curative resection. The TBS is a promising prognostic index in patients with cHCC-CCA. Elevated TBS was related to a lower long-term survival rate and was identified as an independent risk factor for poor DFS and OS. Further research is needed to verify our results.

## Introduction

Combined hepatocellular-cholangiocarcinoma (cHCC-CCA) is a rare subtype that accounts for less than 5% of all primary liver cancers (1). Histologically, cHCC-CCA exhibits both hepatocytic and biliary differentiation. The prognosis of cHCC-CCA is generally worse than that of hepatocellular carcinoma (HCC) and similar to that of intrahepatic cholangiocarcinoma (ICC) (2). Among various therapeutic strategies, surgical resection remains the only curative option for patients with cHCC-CCA (3). However, the 5-year tumor relapse rate exceeds 80% after hepatectomy, and the 5-year overall survival (OS) rate was less than 30% (4).

Conventionally, the tumor-node-metastasis staging system is applied for the prognostic classification of patients with solid malignancies (5, 6). To date, the American Joint Committee on Cancer (AJCC) staging system is the most widely used for the clinical classification of cHCC-CCA (7). In the eighth edition, the T1 category was reclassified using a maximum tumor size of 5 cm, emphasizing the effect of tumor size on outcomes. Moreover, tumor multifocality exerts an equivalent prognostic effect to macrovascular invasion (8).

Recently, a new metric called “tumor burden score (TBS)”, calculated on the basis of tumor size and tumor number, was proposed for risk stratification in multifocal tumors (9). Emerging evidence has shown the promising potential of TBS in stratifying the prognosis of patients with colorectal liver metastasis, HCC, and ICC who underwent surgical resection (10–13). Nevertheless, the prognostic value of TBS in patients with cHCC-CCA has not been evaluated. The present study aimed to investigate the prognostic significance of TBS in surgically treated patients with cHCC-CCA and to compare its predictive accuracy with the other prognostic factors.

## Methods

### Patients

This study was approved by the Ethics Committees of the relevant institutions and was performed in accordance with the Declaration of Helsinki (14). Surgically treated patients with cHCC-CCA from the Seventh Affiliated Hospital of Sun Yat-Sen University and the Third Affiliated Hospital of Sun Yat-Sen University between June 2013 and December 2019 were retrospectively reviewed. The diagnosis was confirmed by pathological examination. The exclusion criteria were as follows: patients with recurrent cHCC-CCA, tumors with positive surgical margin, tumors with local organ invasion, patients who did not undergo resection with curative intent, and those with incomplete clinical data. The included patients were reviewed for basic information, laboratory parameters, and histological and gross tumor features. In addition, all patients were required to sign a consent form for clinical research prior to hepatectomy. The patients were randomly divided into the training cohort and the validation cohort using 2:1 patient matching.

### Follow-up

Tumor markers and contrast-enhanced ultrasonography were performed every month for the first 3 months following surgery, then every 3 months for 1 year, and every half year thereafter. For patients who opted not to go back to the hospital for re-examination, a telephone follow-up survey was carried out. The patients were followed until December 2021 or death. OS was calculated from the date of hepatic resection to the date of the last follow-up or death. In contrast, disease-free survival (DFS) was defined as the interval between the date of hepatic resection and the earliest evidence of recurrence or last follow-up.

### TBS evaluation

TBS was defined as previously reported (9), using the formula: TBS^2^ = (maximum tumor diameter)^2^ + (lesion number)^2^. The maximum tumor diameter and lesion number were obtained from preoperative contrast-enhanced CT scan examination and confirmed by the final pathological report. The optimal cutoff value of TBS was determined by X-tile (version 3.6.1, Yale University) (15). Patients were categorized into the high-TBS group and the low-TBS group according to the cutoff value. The ability of TBS to predict prognosis was validated using the internal validation cohort.

### Statistical analysis

Variables were presented as frequency (%), and continuous variables were compared using the student’s t-test or Wilcoxon rank sum test. Categorical variables were analyzed by the chi-square or Fisher’s exact test, as appropriate. Bivariate survival analyses were performed using Kaplan–Meier curves, and their differences were tested by log-rank test. Cox proportional hazard models (enter method) were employed to assess the potential independent prognostic risk factors and to present adjusted hazard ratio. Variables that were statistically significant in univariate analyses (P-value < 0.05) were entered into multivariate analyses. Adjusted hazard ratios identified by multivariate analyses exhibited risk ratios for tumor relapse or death. However, tumor size and tumor number were excluded from multivariate analyses to avoid collinearity bias (16). The areas under the receiver operator characteristic curve were used to evaluate the predictive accuracy of the significant indicators identified in multivariate analyses. All analyses were performed by MedCalc (version 20.0.3.0, Ostend, Belgien) and SPSS (version 24.0, Chicago, IL, United States). A P-value < 0.05 was considered statistically significant.

## Results

### Patient characteristics

**Figure 1:**
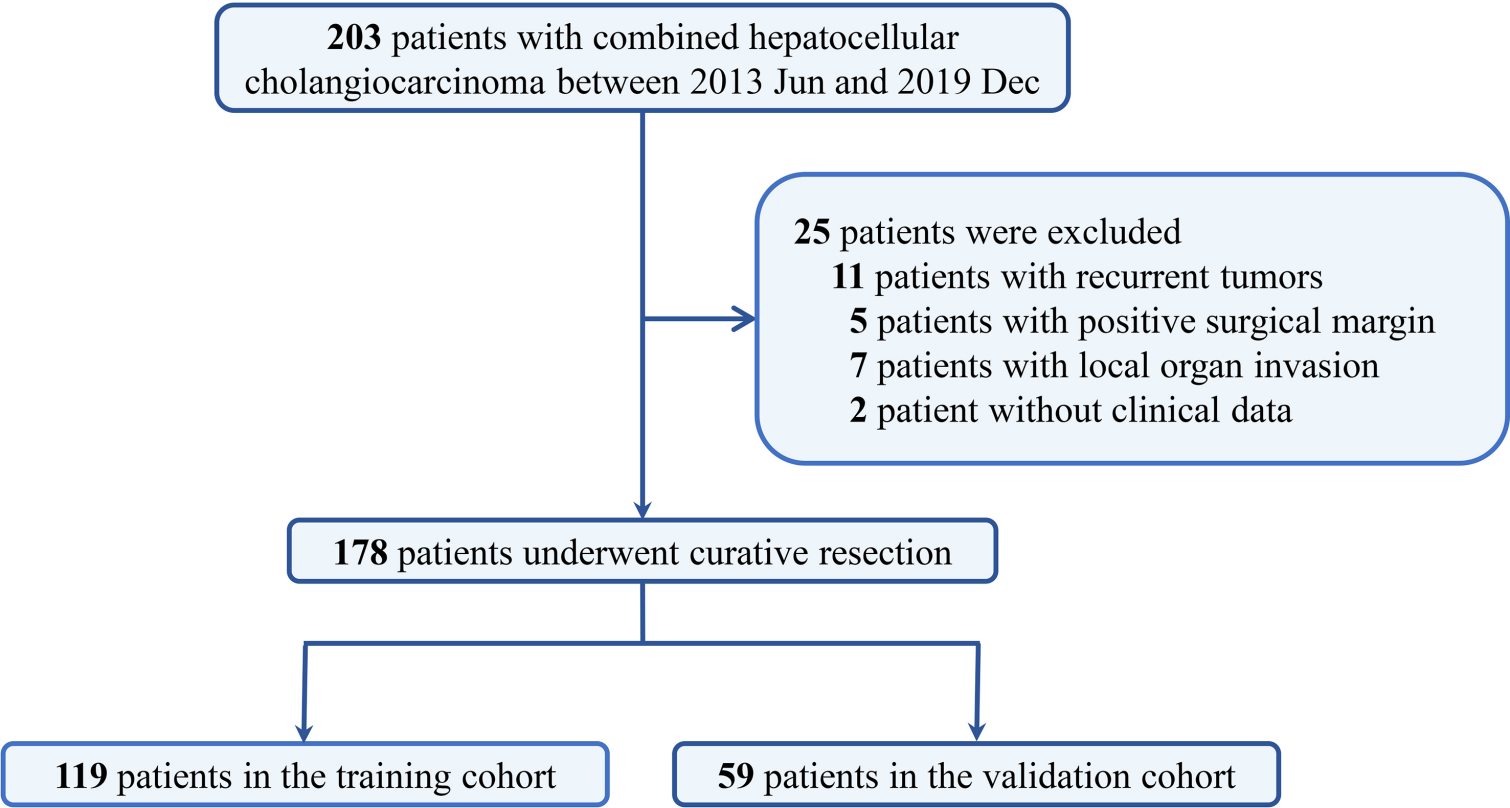
The selection diagram of combined hepatocellular-cholangiocarcinoma patients.

A total of 203 patients who underwent hepatic resection with curative intent between January 2012 and December 2019 were pathologically diagnosed as cHCC-CCA. Among them, 11 patients were associated with recurrent tumors, five patients with positive surgical margins, seven patients with local organ invasion, and two patients had incomplete clinical data. Finally, 178 patients (150 male patients and 128 female patients) were included in the present analysis (119 in the training cohort and 59 in the validation cohort), as shown in **Figure 1**. Among the enrolled patients, 142 (79.8%) were aged less than 60 years, 77 (43.3%) exhibited a maximum tumor diameter of less than 5 cm, and 104 (58.4%) had solitary tumors. No significant difference in baseline characteristics was observed between the training and validation cohorts (**Table 1**).

**Table 1:** Baseline patient characteristics between training and validation cohort.

Variables	All patients (*n* = 178)	Training cohort (*n* = 119)	Validation cohort (*n* = 59)	P-value
Age				0.553
<60	142 (79.8)	93 (78.2)	49 (83.1)	
≥60	36 (20.2)	26 (21.8)	10 (16.9)	
Sex				0.386
Male	150 (84.3)	98 (82.4)	52 (88.1)	
Female	28 (15.7)	21 (17.6)	7 (11.9)	
AFP, ng/ml				0.867
<8	61 (34.3)	40 (33.6)	21 (35.6)	
≥8	117 (65.7)	79 (66.4)	38 (64.4)	
CA19-9 value, U/ml				0.872
<37	78 (43.8)	53 (44.5)	25 (42.4)	
≥37	93 (54.4)	66 (55.5)	34 (57.6)	
HBsAg, +/−				0.621
Positive	116 (65.2)	76 (63.9)	40 (67.8)	
Negative	62 (34.8)	43 (36.1)	19 (32.2)	
Cirrhosis				0.875
Positive	83 (46.6)	56 (47.1)	27 (45.8)	
Negative	95 (53.4)	63 (52.9)	32 (54.2)	
Tumor size, cm				0.842
<5	77 (43.3)	52 (43.7)	25 (42.4)	
≥5	101 (56.7)	67 (56.3)	34 (57.6)	
Tumor number				0.757
Solitary	104 (58.4)	71 (59.7)	33 (55.9)	
Multiple	74 (41.6)	48 (40.3)	26 (44.1)	
Differentiation				0.642
Well	28 (15.7)	19 (16.0)	9 (15.3)	
Moderate-poor	150 (84.3)	100 (84.0)	50 (84.7)	
Capsular invasion				0.741
Positive	113 (63.5)	77 (64.7)	36 (61.0)	
Negative	65 (36.5)	42 (35.3)	23 (39.0)	
MVI				0.730
Positive	51 (28.7)	32 (26.9)	19 (32.2)	
Negative	127 (70.8)	87 (72.3)	40 (67.8)	
Lymph node invasion				0.878
Positive	19 (10.7)	13 (10.9)	6 (10.2)	
Negative	159 (89.3)	106 (89.1)	53 (89.8)	
Adjuvant chemotherapy				0.423
Yes	52 (29.2)	40 (33.6)	12 (20.3)	
No	109 (61.2)	67 (56.3)	42 (71.1)	
Unknown	17 (9.6)	12 (10.1)	5 (8.6)	
TBS grade				0.151
Low	74 (41.6)	54 (45.4)	20 (33.9)	
High	104 (58.4)	65 (54.6)	39 (66.1)	

AFP, alpha-fetoprotein; CA19-9, carbohydrate antigen 19-9; MVI, microvascular invasion; TBS, tumor burden score.

### Association between TBS and clinicopathologic features

The optimal cutoff value of TBS was identified as 5.2 after calculating by using the X-tile (the detailed information was shown in **Supplementary Figures 1, 2**). In the training cohort, 54 (45.4%) patients were classified into the low-TBS group, and 65 (54.6%) were classified into the high-TBS group. The mean TBS value was 3.77 in the low-TBS group. Patients in the high-TBS group were associated with greater frequency of capsular invasion (P-value = 0.012) and lymph node invasion (P-value = 0.036) (**Table 2**).

**Table 2:** Correlation between TBS grade and clinicopathological characteristics in training cohort.

Variables	Low-TBS grade (*n* = 54)	High-TBS grade (*n* = 65)	P-value
Age			0.377
<60	40 (74.1)	53 (81.5)	
≥60	14 (25.9)	12 (18.5)	
Sex			0.815
Male	45 (83.3)	53 (81.5)	
Female	9 (16.7)	12 (18.5)	
AFP, ng/ml			0.079
<8	23 (42.6)	17 (26.2)	
≥8	31 (57.4)	48 (73.8)	
CA19-9 value, U/ml			0.272
<37	29 (53.7)	26 (40.0)	
≥37	25 (46.3)	39 (60.0)	
HBsAg, +/−			0.187
Positive	38 (70.4)	38 (58.5)	
Negative	16 (29.6)	27 (41.5)	
Cirrhosis			0.202
Positive	29 (53.7)	27 (41.5)	
Negative	25 (46.3)	38 (58.5)	
Tumor size, cm			<0.001*
<5	51 (94.4)	1 (1.5)	
≥5	3 (5.6)	64 (98.5)	
Tumor number			0.005*
Solitary	40 (74.1)	31 (47.7)	
Multiple	14 (25.9)	34 (52.3)	
Differentiation			0.924
Well	9 (16.7)	10 (15.4)	
Moderate-poor	45 (83.3)	55 (84.6)	
Capsular invasion			0.012*
Positive	28 (51.9)	49 (75.4)	
Negative	26 (48.1)	16 (24.6)	
MVI			0.154
Positive	11 (20.4)	21 (32.3)	
Negative	43 (79.6)	44 (67.7)	
Lymph node invasion			0.036*
Positive	2 (3.7)	11 (16.9)	
Negative	52 (96.3)	54 (83.1)	
Adjuvant chemotherapy			0.521
Yes	17 (31.5)	23 (35.4)	
No	32 (59.3)	35 (53.8)	
Unknown	5 (9.2)	7 (10.8)	
TBS value	3.77 (0.94)	9.15 (2.38)	<0.001*

AFP, alpha-fetoprotein; CA19-9, carbohydrate antigen 19-9; MVI, microvascular invasion; TBS, tumor burden score; TBS value was showed as mean (standard deviation); *statistically significant.

The association between TBS and clinicopathological features was verified in the validation cohort (**Supplementary Table 1**). In brief, 20 (33.9%) patients were classified into the low-TBS group, whereas 39 (66.1%) patients were classified into the high-TBS group. The frequency of capsular invasion, lymph node invasion, and microvascular invasion MVI was comparable between the two groups.

Among the enrolled patients, three (1.7%) patients received neoadjuvant therapy, and 52 (29.2%) patients were treated with adjuvant chemotherapy. As displayed in **Table 1**, no significant difference was found between the derivation and validation cohorts. Stratified by TBS, postoperative chemotherapy was comparable between the two groups (P-value = 0.521, **Table 2**). In addition, univariate analyses identified that adjuvant chemotherapy might not be a prognostic factor for OS and DFS (**Table 3**). Our results were consistent with previous studies, showing that adjuvant treatment did not influence survival outcomes (17). Collectively, these results implied that cHCC-ICC should be considered as a distinct entity requiring specific therapeutic strategies, especially adjuvant treatments after curative resection.

**Table 3:** Identification of prognostic factors for overall survival and disease-free survival in the training cohort.

Variables	Overall survival				Disease-free survival			
	Univariate		Multivariate		Univariate		Multivariate	
	HR (95% CI)	P-value	HR (95% CI)	P-value	HR (95% CI)	P-value	HR (95% CI)	P-value
Sex (F/M)	0.647 (0.331–1.266)	0.204			0.935 (0.558–1.569)	0.800		
Age (≥60/<60)	1.325 (0.711–2.471)	0.376			1.429 (0.853–2.395)	0.175		
AFP (≥8/<8)	1.525 (0.914–2.544)	0.106			1.714 (1.093–2.689)	0.019*	1.436 (0.892–2.310)	0.136
CA19–9 (≥37/<37)	1.325 (0.817–2.151)	0.254			1.479 (0.967–2.262)	0.071		
HBsAg	1.244 (0.750–2.064)	0.397			1.208 (0.782–1.865)	0.394		
Cirrhosis	1.118 (0.700–1.784)	0.641			1.132 (0.751–1.705)	0.555		
Tumor size (≥5/<5)	1.003 (0.624–1.613)	0.991			1.597 (1.056–2.416)	0.027*		
Tumor number (multiple/solitary)	1.189 (0.724–1.950)	0.494			1.751 (1.154–2.656)	0.006*		
Differentiation (moderate-poor/well)	1.111 (0.399–1.648)	0.563			1.131 (0.512–2.495)	0.761		
Liver capsule invasion	1.166 (0.719–1.892)	0.534			1.358 (0.879–2.098)	0.168		
MVI	1.977 (1.206–3.242)	0.007*	2.289 (1.374–3.813)	0.001*	1.660 (1.063–2.592)	0.026*	1.654 (1.054–2.597)	0.029*
Lymph node invasion	2.727 (1.386–5.366)	0.004*	2.546 (1.255–5.164)	0.010*	1.695 (1.124–2.557)	0.012*	1.576 (1.006–2.469)	0.047*
Adjuvant chemotherapy	0.821 (0.534–1.342)	0.556			0.751 (0.512–1.273)	0.329		
TBS grade (high/low)	2.510 (1.524–4.133)	<0.001*	2.361 (1.417–3.934)	<0.001*	3.177 (2.040–4.949)	<0.001*	2.643 (1.629–4.288)	<0.001*

M, male; F, female; MVI, microvascular invasion; CA19-9, carbohydrate antigen 19-9; HR, hazard ratio; CI, confidence interval; *statistically significant.

### Association between TBS and patient prognosis

**Figure 2:**
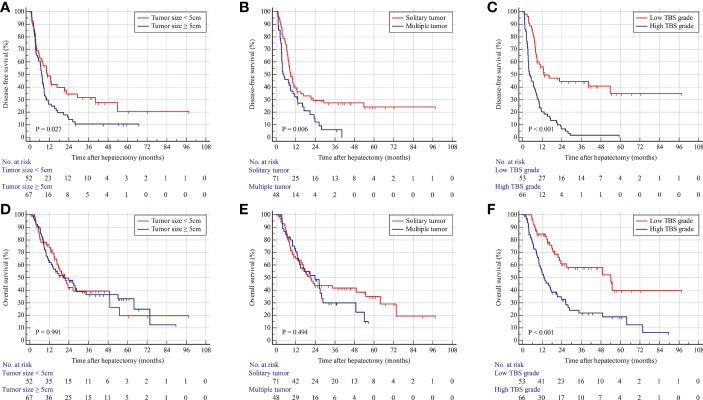
Kaplan–Meier curves for DFS and OS stratified by tumor size **(A, D)**, tumor number **(B, E)**, and TBS grade **(C, F)** in the derivation cohort.

As shown in **Figure 2**, patients with a maximum tumor diameter of less than 5 cm were associated with longer DFS but similar OS compared with those with tumor size larger than 5 cm (**Figures 2A, D**). In addition, patients with solitary tumors were associated with longer DFS but similar OS compared with those with multiple tumors (**Figures 2B, E**). The patients were stratified into two groups on the basis of TBS. The patients in the low-TBS group were associated with better OS and a lower rate of tumor relapse compared with those with high TBS (**Figures 2C, F**). The Cox regression models indicated that high TBS was an independent risk factor for poor OS [hazard ratio (HR) = 2.361; 95% confidence interval (CI), 1.417–3.934; P-value < 0.001] and DFS (HR = 2.643; 95% CI, 1.629–4.288; P-value < 0.001) (**Table 3**). Furthermore, MVI and lymph node invasion were also independent prognostic indicators for poor OS and DFS.

**Figure 3:**
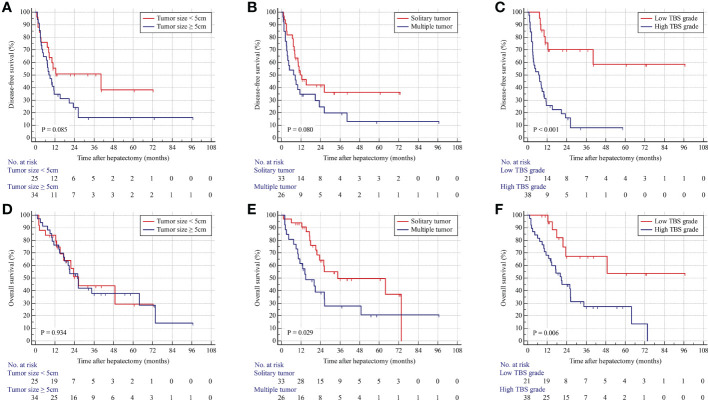
Kaplan–Meier curves for DFS and OS stratified by tumor size **(A, D)**, tumor number **(B, E)**, and TBS grade **(C, F)** in the validation cohort.

In the internal validation cohort, patients with solitary tumors were associated with better OS and similar DFS compared with those with multiple tumors. Patients in the low-TBS group were associated with better prognoses. Consistently, Cox regression models identified high TBS as an independent prognostic indicator for poor OS and DFS (**Figure 3**; **Supplementary Table 2**).

The predictive accuracy of TBS was compared with tumor size, tumor number, MVI, and lymph node invasion. As shown in **Supplementary Table 3**, TBS showed the highest area under curve (AUC) in predicting OS (0.689; 95% CI, 0.584–0.782) and DFS (0.772; 95% CI, 0.672–0.853), indicating that TBS was the most effective in predicting patient prognosis. Subsequently, the specificity and sensitivity of these indicators were compared. In addition, the AUCs of TBS based on those previously reported cutoff values were compared, revealing that the present TBS cutoff value was the most accurate in predicting long-term outcomes for patients with cHCC-ICC (**Supplementary Table 4**).

## Discussion

Epidemiologically, cHCC-CCA is a rare subtype of primary liver cancer, accounting for less than 5% of the cases (1). cHCC-ICC may exhibit both hepatocytic and biliary differentiation (2). The likelihood of viral hepatitis in patients with cHCC-ICC is intermediate between HCC and ICC (18, 19). In addition, cHCC-ICC cells may produce AFP and CA19-9. Clinically, a simultaneous increase in serum levels of both markers strongly suggests the diagnosis of cHCC-ICC. Nevertheless, only a minority of patients were associated with an increase in both serum markers. The clinical symptoms of cHCC-ICC are most often associated with advanced tumoral disease and are not apparent in the early stage. Therefore, more than half of the patients were diagnosed at advanced stages. Currently, there is no therapeutic guideline for cHCC-CCA, and curative resection is considered the most effective treatment (20). However, the long-term outcome of patients with cHCC-CCA is still poor due to rapid progression and frequent tumor relapse. Improving the prognosis of patients with cHCC-CCA remains a medical challenge. The present study demonstrated that TBS, calculated on the basis of the maximum tumor diameter and the number of lesions, was a stable and independent prognostic indicator for both DFS and OS in patients with cHCC-CCA who undergoing radical hepatic resection.

The staging strategy for cHCC-CCA was first incorporated into the ICC-tumor-node-metastasis (TNM) staging system in the seventh edition of the AJCC staging manual (21), in which tumor size was not included as a factor. However, in the eighth edition, the T1 stage was stratified into T1a and T1b on the basis of tumor size with a cutoff value of 5 cm. T2a and T2b were merged into T2, which represented the equivalent effect of tumor multifocality and vascular invasion (8). A growing number of studies have identified tumor size as an independent prognostic predictor for poor survival outcomes in patients with cHCC-CCA (22, 23). Our results in this study reflected the previous findings that patients with tumor size > 5 cm were associated with faster tumor relapse. However, the prognostic value of the number of lesions in patients with cHCC-CCA has been controversial. Kim et al. suggested that patients with solitary tumors were associated with a superior prognosis than those with multiple lesions (24), whereas Jiang et al. demonstrated that the tumor number was not a prognostic indicator for cHCC-CCA after curative resection (25). Our results revealed that multiple tumor lesions were associated with worse DFS but similar OS compared with those with solitary lesions. Nevertheless, the prognostic value of tumor size and tumor number in patients with cHCC-CCA should be confirmed by future studies with larger sample sizes.

TBS was defined as the distance from the origin of a Cartesian plane using maximum tumor size as the X-axis and tumor number as the Y-axis (9). As previously reported, TBS is a valuable tool in evaluating prognosis for colorectal liver metastases, HCC, and ICC (13, 26, 27). The present study assessed the significance of TBS in dictating the prognosis of patients with cHCC-CCA after curative liver resection. Elevated TBS grade was associated with poor DFS and OS and was identified as a stable, independent prognostic indicator for long-term outcomes. Notably, the predictive effect of TBS outperformed tumor size and number alone and MVI and lymph node invasion in evaluating DFS and OS.

The following limitations should be considered when interpreting our results. First, this was a retrospective study with a limited number of cases, which might involve selection bias or unmeasured confounding factors. Second, although the cutoff value for TBS was validated by our internal validation cohort, its applicability to overseas patient populations remains unknown. In addition, as only surgically treated patients with cHCC-CCA (a limited part of the overall population of patients with cHCC-CCA) were included in the analyses, the results are only applicable to patients who undergoing curative resection.

In conclusion, this study suggests the significance of tumor morphology in assessing the prognosis of patients with cHCC-CCA who undergoing curative resection. The TBS, based on the maximum tumor diameter and lesion number, is a promising index in the prognostic evaluation of patients with cHCC-CCA. Elevated TBS was associated with worse long-term survival and was identified as an independent risk factor for poor DFS and OS. Nevertheless, these results should be verified by further studies.

## Data availability statement

The original contributions presented in the study are included in the article/**Supplementary Material**. Further inquiries can be directed to the corresponding authors.

## Ethics statement

The studies involving human participants were reviewed and approved by Seven Affiliated Hospital of Sun Yat-sen University. The patients/participants provided their written informed consent to participate in this study.

## Author contributions

Conceptualization: GD, TZ, and DT. Data curation: GD, Z-BC, and Y-WF. Formal analysis: GD, J-KR, and Y-JT. Supervision: TZ and DT. Writing—original draft: GD, J-KR, and H-TW. All the authors approved the final version of manuscript.
