# Supplementary material for: Tumor burden score dictates prognosis of patients with combined hepatocellular cholangiocarcinoma undergoing hepatectomy
Source: Front Oncol. 2023 Jan 23;12:977111. doi: 10.3389/fonc.2022.977111 (PMC9900097; doi:10.3389/fonc.2022.977111)
Supplement: Supplementary file 1 [file DataSheet_1.pdf]

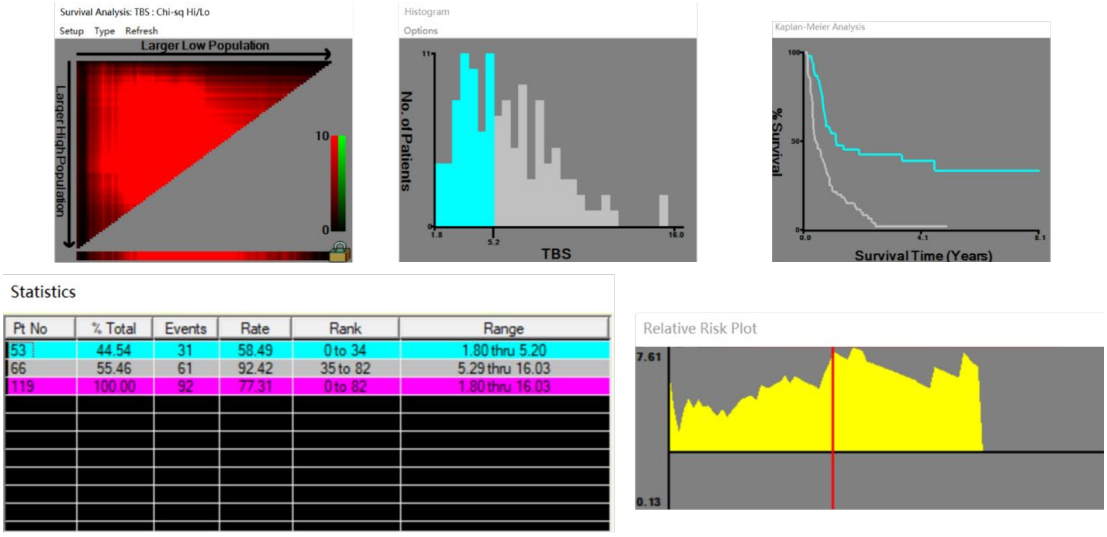

Pt No	% Total	Events	Rate	Rank	Range
53	44.54	31	58.49	0 to 34	1.80 thru 5.20
66	55.46	61	92.42	35 to 82	5.29 thru 16.03
119	100.00	92	77.31	0 to 82	1.80 thru 16.03

Supplementary figure 1. The status of tumor relapse was set as endpoint and disease-free survival was set as survival time. The optimal cut-off value of TBS was identified as 5.2.

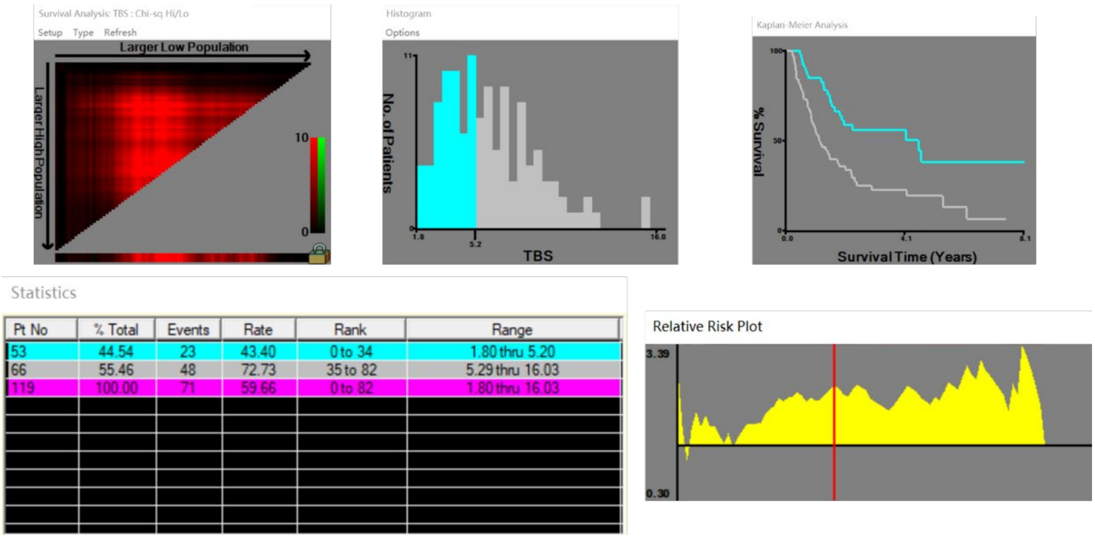

Pt No	% Total	Events	Rate	Rank	Range
53	44.54	23	43.40	0 to 34	1.80 thru 5.20
66	55.46	48	72.73	35 to 82	5.29 thru 16.03
119	100.00	71	59.66	0 to 82	1.80 thru 16.03

Supplementary figure 2. The status of survival was set as endpoint and overall survival was set as survival time. The optimal cut-off value of TBS was also identified as 5.2.

Supplementary table 1. Correlation between TBS grade and clinicopathological characteristics in validation cohort.

Variables	Low TBS grade (*n*=21)	High TBS grade (*n*=38)	P value
Age			0.737
< 60	18 (85.7)	31 (81.6)	
≥ 60	3 (14.3)	7 (18.4)	
Sex			0.679
Male	19 (90.5)	33 (86.8)	
Female	2 (9.5)	5 (13.2)	
AFP, ng/ml			0.169
< 8	10 (47.6)	11 (28.9)	
≥ 8	11 (52.4)	27 (71.1)	
CA19-9 value, U/ml			0.275
< 37	11 (52.4)	14 (36.8)	
≥ 37	10 (47.6)	24 (63.2)	
HBsAg, +/-			0.149
Positive	17 (81.0)	23 (60.5)	
Negative	4 (19.0)	15 (39.5)	
Cirrhosis			0.790
Positive	9 (42.9)	18 (47.4)	
Negative	12 (57.1)	20 (52.6)	
Tumor size, cm			<0.001*
< 5	18 (85.7)	7 (18.4)	
≥ 5	3 (14.3)	31 (81.6)	
Tumor number			0.029*
Solitary	16 (76.2)	17 (44.7)	
Multiple	5 (23.8)	21 (55.3)	
Differentiation,			0.452
Well	4 (19.0)	5 (13.2)	
Moderate-poor	17 (81.0)	33 (86.8)	
Capsular invasion			0.405
Positive	11 (52.4)	25 (65.8)	
Negative	10 (47.6)	13 (34.2)	
MVI			0.149
Positive	4 (19.0)	15 (39.5)	
Negative	17 (81.0)	23 (60.5)	
Lymph node invasion			0.407
Positive	1 (4.8)	5 (13.2)	
Negative	20 (95.2)	33 (86.8)	
Adjuvant chemotherapy			0.752
Yes	5 (23.8)	7 (18.4)	
No	14 (66.7)	28 (73.7)	
Unknown	2 (9.5)	3 (7.9)	

TBS value	4.16 (1.97)	9.05 (3.47)	<0.001*

AFP, alpha-fetoprotein; CA19-9, carbohydrate antigen 19-9; MVI, microvascular invasion; TBS, tumor burden score; TBS value was showed as mean (standard deviation); *statistically significant.

Supplementary table 2. Identification of prognostic factors for overall survival and disease-free survival in the validation cohort.

Variables	Overall survival				Disease-free survival			
	Univariate		Multivariate		Univariate		Multivariate	
	HR (95%CI)	P value	HR (95%CI)	P value	HR (95%CI)	P value	HR (95%CI)	P value
Sex (F/M)	0.904 (0.317-2.584)	0.851			0.857 (0.333-2.209)	0.750		
Age (≥60/<60)	1.374 (0.524-3.606)	0.518			0.662 (0.259-1.695)	0.390		
AFP (≥8/<8)	2.003 (0.902-4.450)	0.088			1.930 (0.962-3.870)	0.064		
CA19-9 (≥37/<37)	1.684 (0.794-3.574)	0.174			1.193 (0.619-2.302)	0.598		
HBsAg	1.366 (0.632-2.951)	0.427			1.191 (0.604-2.347)	0.614		
Cirrhosis	1.735 (0.863-3.486)	0.122			1.458 (0.783-2.713)	0.235		
Tumor size (≥5/<5)	1.031 (0.503-2.111)	0.934			1.735 (0.927-3.247)	0.085		
Tumor number (multiple/solitary)	2.221 (1.086-4.544)	0.029*			1.769 (0.934-3.352)	0.080		
Differentiation (moderate-poor/well)	2.129 (0.689-6.581)	0.189			1.935 (0.694-5.394)	0.207		
Liver capsule invasion	2.006 (0.946-4.254)	0.070			1.254 (0.659-2.386)	0.490		
MVI	1.106 (0.553-2.292)	0.787			1.388 (0.720-2.676)	0.328		
Lymph node invasion	5.190 (1.858-14.492)	0.002*	5.214 (1.824-14.905)	0.002*	2.614 (1.007-6.787)	0.048*	1.925 (0.739-5.015)	0.180
Adjuvant chemotherapy	0.863 (0.586-1.424)	0.742			0.743 (0.456-1.363)	0.421		
TBS grade (high/low)	3.264 (1.343-7.933)	0.006*	3.250 (1.335-7.912)	0.009*	5.194 (2.261-11.930)	<0.001*	4.965 (2.151-11.460)	<0.001*

M, male; F, female; MVI, microvascular invasion; CA19-9, carbohydrate antigen 19-9; HR, hazard ratio; CI, confidence interval; *statistically significant.

Supplementary table 3. Comparison of predictive value in OS and DFS.

	Accuracy	95% CI	Specificity (%)	Sensitivity (%)	P-value
OS					
-TBS grade	0.689	0.584-0.782	64.58	74.21	Ref.
-Tumor number	0.519	0.412-0.625	41.67	60.42	0.020*
-Tumor size	0.512	0.405-0.618	45.83	57.14	0.037*
-Lymph node invasion	0.581	0.473-0.684	93.75	14.29	0.040*
-MVI	0.585	0.477-0.688	83.33	34.78	0.101
DFS					
-TBS grade	0.772	0.672-0.853	87.42	67.03	Ref.
-Tumor number	0.556	0.448-0.660	74.07	45.05	<0.001*
-Tumor size	0.611	0.503-0.711	62.96	61.96	0.008*
-Lymph node invasion	0.554	0.446-0.658	11.11	89.01	<0.001*
-MVI	0.572	0.448-0.660	85.19	31.11	0.001*

OS, overall survival; DFS, disease-free survival; TBS, tumor burden score; MVI, microvascular invasion; *statistically significant.

Supplementary table 4. Comparison of predictive value in OS and DFS using different cut-off values.

	Accuracy	95% CI	P-value
OS			
-TBS grade (5.2)	0.689	0.584-0.782	Ref.
-TBS grade (3)	0.501	0.395-0.606	<0.001*
-TBS grade (3.36)	0.537	0.431-0.641	0.009*
-TBS grade (4.71)	0.657	0.550-0.754	0.481
DFS			
-TBS grade (5.2)	0.772	0.672-0.853	Ref.
-TBS grade (3)	0.547	0.440-0.651	0.004*
-TBS grade (3.36)	0.610	0.503-0.709	0.023*
-TBS grade (4.71)	0.696	0.592-0.787	0.621

OS, overall survival; DFS, disease-free survival; TBS, tumor burden score; *Statistical significance.
